# miR-124a and miR-155 enhance differentiation of regulatory T cells in patients with neuropathic pain

**DOI:** 10.1186/s12974-016-0712-6

**Published:** 2016-09-20

**Authors:** Jens Heyn, Benjamin Luchting, Ludwig C. Hinske, Max Hübner, Shahnaz C. Azad, Simone Kreth

**Affiliations:** Department of Anesthesiology, Ludwig-Maximilians University Munich, Marchioninistr. 15, 81377 Munich, Germany

**Keywords:** Neuropathic pain, miRNA, Regulatory T cells, Histone deacetylase sirtuin1, Analgesia

## Abstract

**Background:**

Accumulating evidence indicates that neuropathic pain is a neuro-immune disorder with enhanced activation of the immune system. Recent data provided proof that neuropathic pain patients exhibit increased numbers of immunosuppressive regulatory T cells (Tregs), which may represent an endogenous attempt to limit inflammation and to reduce pain levels. We here investigate the molecular mechanisms underlying these alterations.

**Methods:**

Our experimental approach includes functional analyses of primary human T cells, 3′-UTR reporter assays, and expression analyses of neuropathic pain patients’ samples.

**Results:**

We demonstrate that microRNAs (miRNAs) are involved in the differentiation of Tregs in neuropathic pain. We identify miR-124a and miR-155 as direct repressors of the histone deacetylase sirtuin1 (SIRT1) in primary human CD4^+^ cells. Targeting of SIRT1 by either specific siRNA or by these two miRNAs results in an increase of Foxp3 expression and, consecutively, of anti-inflammatory Tregs (siRNA: 1.7 ± 0.4; miR-124a: 1.5 ± 0.4; miR-155: 1.6 ± 0.4; *p* < 0.01). As compared to healthy volunteers, neuropathic pain patients exhibited an increased expression of miR-124a (2.5 ± 0.7, *p* < 0.05) and miR-155 (1.3 ± 0.3; *p* < 0.05) as well as a reduced expression of SIRT1 (0.5 ± 0.2; *p* < 0.01). Moreover, the expression of these two miRNAs was inversely correlated with SIRT1 transcript levels.

**Conclusions:**

Our findings suggest that in neuropathic pain, enhanced targeting of SIRT1 by miR-124a and miR-155 induces a bias of CD4^+^ T cell differentiation towards Tregs, thereby limiting pain-evoking inflammation. Deciphering miRNA-target interactions that influence inflammatory pathways in neuropathic pain may contribute to the discovery of new roads towards pain amelioration.

**Trial registration:**

German Clinical Trial Register DRKS00005954

**Electronic supplementary material:**

The online version of this article (doi:10.1186/s12974-016-0712-6) contains supplementary material, which is available to authorized users.

## Background

Neuropathic pain is caused by impairment of somatosensory functions in both the peripheral and central nervous system [1]. It is often associated with spontaneous pain, dysesthesia, paraesthesia, and hyperalgesia (increased pain caused by painful stimuli) and allodynia (increased pain caused by non-painful stimuli) [2, 3]. The treatment of neuropathic pain is ambitious, and outcomes often are unsatisfactory [4]. Despite intensive analgesic treatment, significant attenuation of pain is only achieved in a limited number of patients [5].

There is emerging evidence that aberrant responses of the immune system substantially contribute to the development of neuropathic pain [6]. Immune cells respond to nerve injury by migration into the nervous system at the side of injury, thereby releasing mediators, which affect intercellular signaling [7]. Although the precise role of immune cells in neuropathic pain remains unclear, adoptive transfer of immune cells producing pro-inflammatory cytokines significantly increase pain sensitivity, whereas transfer of cells producing anti-inflammatory cytokines decrease pain sensitivity in nerve-injured rats [8]. Recent data investigating neuropathic pain in humans published by our group point into the same direction. We showed that patients exhibit altered ratios of peripheral T-helper cell subsets. Specifically, increased numbers of immunosuppressive regulatory T cells (Tregs) have been found [9, 10]. This could reflect an endogenous strategy to limit inflammation and to reduce pain levels in neuropathic pain, which is of interest with respect to future treatment approaches.

The current study aims to investigate the molecular mechanisms underlying these alterations. We focused on the expression of the histone deacetylase sirtuin1 (SIRT1), which is supposed to play a significant role in the development and function of Tregs [11, 12]. SIRT1 controls transcription factor forkheadbox-p3 (Foxp3), the master regulator of Treg differentiation [12]. Treatment with SIRT1 inhibitors increased Foxp3 gene expression with consecutive increase of Treg differentiation in mice [13]. We thus hypothesized that these mechanisms might also be involved in the Treg alterations observed in neuropathic pain patients.

## Methods

### Patients

Patients appearing with neuropathic pain in our Department of Pain Medicine were verified for fulfilling the inclusion criteria and asked about their agreement to participate in the study. Neuropathic pain was defined as “pain caused by a lesion or disease of the somatosensory nervous system” such as polyneuropathy, postherpetic neuralgia, or trigeminal neuralgia/neuropathy using the PainDETECT-questionnaire [14]. Additionally, quantitative sensory testing was performed to all patients, according to the protocol of the German Research Group on neuropathic pain [15]. Patients suffering from low back pain with radiculopathy (even if the radicular component was clearly predominant) or patients with autoimmune, chronic, inflammatory, neoplastic, or psychiatric diseases were excluded. None of the patients had been treated with corticosteroids or had received known immunomodulatory agents currently or in the past. Acute inflammation was excluded by determination of C-reactive protein (CRP), total- and differential leucocyte, and measurement of body temperature. Eleven patients fulfilled the inclusion criteria. Blood samples of these patients were obtained as well as from 9 healthy volunteers after written consents were obtained. Additionally, patients were asked to quote their average pain intensity using an 11-point numerical rating scale (NRS) with 0 representing “no pain” and 10 “worst pain imaginable”. For patients’ characteristics, see Table 1.

**Table 1:** Patient characteristics

Item	Healthy	Neuropathic pain	*p* value
Numbers (*n*)	9	11	
Age	36 ± 9	54 ± 12	< 0.05
Female	55 %	64 %	n.s.
BMI	22.9 ± 2.9	25.3 ± 3.4	n.s.
NRS (rest)	0.0 ± 0.0	4.8 ± 2.3	< 0.05
NRS (motion)	0.0 ± 0.0	7.6 ± 1.7	< 0.05
KAB	1.5 ± 0.4	3.4 ± 0.7	< 0.05

Results are expressed as mean ± standard deviation (SD)

*BMI* body mass index, *NRS (rest/motion)* numeric rating scale (0 to 10) of pain, 0: “no pain,” 10: “worst pain imaginable,” *KAB* questionnaire for self-perceived stress ranging (1–6), 1: “no stress,” 6: “maximum stress,” *n.s.* not significant

The prospective study protocol followed the principles of the Declaration of Helsinki and was reviewed and approved by the Ethics Committee of the LMU Munich and registered on German Clinical Trial Register (Registration Trial DRKS00005954). Patients included in this study have also been part of a recently published study by Luchting et al. [9] showing an anti-inflammatory T cell shift in patients suffering from neuropathic pain.

### miRNA selection and target prediction

In the current manuscript, we focused on the evaluation of miR-124a and miR-155. These microRNAs (miRNAs) were selected as follows: We first sought to identify miRNAs that have been found to be differentially expressed in pain- and inflammation-related syndromes [16, 17]. Of these, only miR-124a and miR-155 were predicted to target SIRT1. These predictions were based on the established target prediction algorithm TargetScan [18].

### RNA isolation and cDNA synthesis

Total RNA was isolated using either the RNAqueous® Micro Kit or the mirVana miRNA Isolation Kit followed by subsequent DNase treatment (Turbo DNase, Ambion) according to the manufacturer’s instructions. Quantity and purity of the isolated RNA were measured using a NanoDrop 2000 spectrophotometer (Thermo Scientific). Complementary DNA (cDNA) was synthesized from 1 μg of total RNA using SuperScript III First Strand Synthesis System (Invitrogen), as per manufacturer’s instructions.

### Quantitative RT-PCR

cDNA was synthesized from equal amounts of total RNA using Superscript III reverse transcriptase (Invitrogen) and oligo(dT) and random hexamer primers following the supplier’s instructions. Quantitative analyses of messenger RNA (mRNA) levels were performed in duplicates on a Light Cycler 480 (Roche Diagnostics) using either UPL probes and specific primers or specific single assays (Table 2, Roche Diagnostics, Penzberg). The cycling conditions comprised an initial denaturation phase at 95 °C for 5 min, followed by 45 cycles at 95 °C for 10 s, 60 °C for 30 s, and 72 °C for 15 s. Data were normalized to the reference genes SDHA and TBP [19].

**Table 2:** Primer sequences for real-time PCR

	Sequence/assay ID
Foxp3	Roche RealTime Ready Single Assay ID 113503
SIRT1	for 5′-TGT ACG ACG AAG ACG ACG AC-3′ (UPL probe #63) rev 5′-TTC ATC ACC GAA CAG AAG GTT-3′ (UPL probe #63)
TBP	for 5′-GAACATCATGGATCAGAACAACA-3′ (UPL probe #87) rev 5′-ATAGGGATTCCGGGAGTCAT-3′ (UPL probe #87)
SDHA	for 5′-GAGGCAGGGTTTAATACAGCA-3′ (UPL probe #80) rev 5′-CCAGTTGTCCTCCTCCATGT-3′ (UPL probe #80)

#### Quantification of miRNA expression

Expression of miR-124a, miR-155, and U47 (endogenous control) was quantified using TaqMan miRNA assays (Applied Biosystems) following the manufacturer’s protocol. In brief, equal amounts of RNA (10 ng) were reverse transcribed using miRNA-specific stem-loop primers and the TaqMan MicroRNA Reverse Transcription Kit (Applied Biosystems). Real-time PCR (RT-PCR) was performed in duplicate using LightCycler 480 Probes Master on the LightCycler 480 instrument applying the following cycling conditions: denaturing at 95 °C for 10 min, 45 cycles of 95 °C for 15 s, and 60 °C for 60 s. U47 RNA was used for normalization of miRNA expression data.

### Western blot analysis

Thirty-five micrograms of total protein extracts was electrophoresed in an 8 % SDS–PAGE and subsequently electroblotted onto PVDF membranes. Non-specific binding sites on the membrane were blocked using 5 % non-fat dry milk in TBS-Tween. SIRT1 antibody (Cell Signaling Technology, Danvers, MA) was diluted in PBST supplemented with 1 % non-fat dry milk (dilution factor 1:2000). β-actine (Cell Signaling Technology, dilution factor 1:40,000) served as a loading control. Immunoreactive bands were visualized using horseradish peroxidase-labeled goat anti-mouse or goat anti-rabbit antibodies and the Signal Fire ECL Substrate (Cell Signaling Technology, Danvers, MA).

### Purification of peripheral human CD4^+^ T cells

CD4^+^ T cells were isolated from peripheral blood mononuclear cells (PBMCs) by magnetic separation with Whole Blood CD4 MicroBeads (MACS Miltenyi Biotec, Bergisch Gladbach, Germany) according to the manufacturer’s instructions. Viability and cell number were ascertained by ViCell analyzer (Beckman Coulter, Fullerton, CA).

### Cell cultures and stimulation conditions

Primary CD4^+^ T cells were cultured in six-well plates in RPMI 1640 medium (Sigma-Aldrich, St. Louis, MO) supplemented with 10 % heat-inactivated fetal calf serum (Biochrom, Berlin, Germany), penicillin (100 IU/mL), streptomycin (100 μg/mL), sodium pyruvate, and l-glutamine (Gibco, Life Technologies, Darmstadt, Germany) at 37 °C in a humidified atmosphere of 5 % CO_2_ in air.

For differentiation into Tregs, CD4^+^ T cells (7 × 10^5^/mL) were cultured under Treg differentiating conditions (anti-CD3/CD28 Dynabeads (Invitrogen, Carlsbad, Germany) for 36 h, rhIL-2 and TGF-ß for four additional days.) To evaluate the effect of miR-124a, miR-155, or siSIRT1 on Treg differentiation, CD4^+^ T cells were transfected with these miRNAs, siRNA, or negative control 6 h before stimulation of CD4^+^ T cells was initiated.

### Flow cytometric staining and analysis

For identification and quantification of Tregs, multicolor flow cytometry was used after surface staining of peripheral blood mononuclear cells with specific antibodies. These antibodies include anti-human CD4 and Foxp3. To quantify the number of Tregs after transfection of CD4^+^ T cells and incubation under Treg skewing conditions, Tregs were identified by surface staining with anti-human CD4+ and intracellular staining with FoxP3 antibody (Biolegend, San Diego, CA, USA). The amount of Tregs was expressed as a ratio of CD4^+^Foxp3^+^ T cells as a percentage of CD4^+^ T cells. Tregs in patients and healthy volunteers were identified after surface staining of PBMCs with monoclonal antibodies specific for anti-human CD4, CD25, and CD127 and intracellular staining with an anti-human Foxp3 antibody. CD4+CD25highCD127lowFoxp3+ cells were defined as Tregs.

### Cloning and mutagenesis of vector constructs

The psiCHECK-2 Target Expression Vector (Promega, Madison, WI, USA) was used for generation of 3′-untranslated region (3′-UTR) reporter constructs as described before [20]. Briefly, the 3′-UTR of SIRT1 containing the predicted target sites of miRNA-124a and miRNA-155 were amplified by PCR from human genomic DNA (100 ng) with the primers given in Table 3 (synthesized by Metabion, Martinsried, Germany). Cycling conditions were as follows: 95 °C for 3 min denaturing; 30 cycles of 95 °C for 30 s, 61.2 °C for 30 s, and 72 °C for 30 s; and a final extension at 72 °C for 5 min. PCR products were cloned into the *PmeI* and *XhoI* restriction sites of the psiCHECK-2 plasmid*.* Site-directed mutagenesis [20] of the putative miR-124a or the three miR-155 binding sites was performed using the QuickChange Lightning Mutagenesis Kit (Stratagene) with the primers given in Table 4. All plasmids were verified by sequence analysis (MWG Biotech, Ebersberg, Germany).

**Table 3:** Primer sequences for the 3′-UTR of SIRT1

Primer	Sequence	Restriction site
SIRT1-3′UTR for	5′-*CTCGAG*CTGTGAAACAGGAAGTAACAGACA-3′	*XhoI*
SIRT1-3′UTR rev	5′-*GTTTAAA* **C**TGGCAGTAATGGTCCTAGCTG-3′	*PmeI*

Restriction enzymes and the cutting sides of these enzymes are italicized

**Table 4:** Primers for mutagenesis

Primer	Sequence	Position
SIRT mut 124a for	5′-TATTTAAAAGCTTAGCCTG*GA*TTAAAACTAGAGATCAACTTTCTCAGA-3′	1211–1217
SIRT mut 124a rev	5′-GCTGAGAAAGTTGATCTCTAGTTTTAA*TC*CAGGCTAAGCTTTTAAATA-3′	
SIRT mut 155_1 for	5′-CAGGAATTGTTCCACCAG*GG*TTAGGAACTTTAGCATGTC-3′	36–42
SIRT mut 155_1 rev	5′-GACATGCTAAAGTTCCTAA*CC*CTGGTGGAACAATTCCTG-3′	
SIRT mut 155_2 for	5′-TTGATCTTTTCCACAAG*GG*TTAAACTGCCAAAATGTG-3′	929–935
SIRT mut 155_2 rev	5′-CACATTTTGGCAGTTTAA*CC*CTTGTGGAAAAGATCAA-3′	
SIRT mut 155_3 for	5′-GAAATTGCACAGTAAG*GG*TTTATTTTTCAGACCATT-3′	1408–1414
SIRT mut 155_3 rev	5′-AATGGTCTGAAAAATAAA*CC*CTTACTGTGCAATTTC-3′	

Sequenzes of the mutagenesis are italicized

### Cell transfections and luciferase assay

Cell transfections were performed by electroporation using the Neon™ transfection system (Invitrogen, Life Technologies, Darmstadt, Germany). CD4^+^ T cells were transfected with 50 nM pre-miR-124a, pre-miR-155, or negative control. For luciferase assay, HEK-293 cells were co-transfected with 1 μg of psiCheck-2 dual luciferase reporter plasmids containing the 3′-UTR of SIRT1 and either pre-miR-124a, pre-miR-155, or negative control (Ambion, Austin, TX, USA) at a final concentration of 50 nM. HEK-293 cells (European Collection of Cell Cultures) were grown in Dulbecco’s modified Eagle medium (DMEM—Lonza, Walkersville, MD) supplemented with 10 % heat-inactivated fetal bovine serum (FBS), 1 % penicillin/streptomycin/glutamine, and 1 % NEAA at 37 °C in a humidified atmosphere of 5 % CO_2_ in air. Forty-eight hours after transfection, cells were lysed and analyzed for firefly and renilla luciferase activity using the Dual-Glo-Luciferase Assay System (Promega), and *Renilla* luciferase activities were normalized to *Firefly* activities. All experiments were performed in triplicates.

### Statistical analyses

All statistical analyses were performed using SigmaStat 12.0 (Systat Software, Chicago, USA). Every statistical analysis was started with testing for normal distribution using the Shapiro-Wilk Test. Further analyses were performed with Student’s *t* test for all data with normal distribution and the nonparametric Mann-Whitney rank-sum test for all data without normal distribution. Values are expressed as mean ± standard deviation (SD). *p* values <0.05 were considered as statistically significant.

## Results

### SIRT1 mRNA expression is decreased in neuropathic pain

**Fig. 1:**
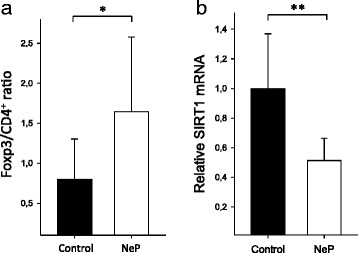
SIRT1 is down-regulated in CD4^+^ T cells of patients with neuropathic pain. **a** T cells obtained from patients with neuropathic pain were characterized by surface staining with monoclonal antibodies specific for anti-human CD4, CD25, and CD127 and intracellular staining with an anti-human Foxp3 antibody followed by flow cytometric analysis. The Foxp3/CD4^+^ ratio found in neuropathic pain patients (NeP, *n* = 11) and in healthy controls (control, *n* = 9) is shown. **b** Total RNA was extracted from purified CD4^+^ T cells of patients with neuropathic pain (NeP, *n* = 11) as well as from CD4^+^ T cells of healthy volunteers (control, *n* = 9). Relative expression of SIRT1 was quantified by quantitative PCR (qPCR) using SDHA and TBP as reference genes. The results indicate fold reduction of SIRT1 in patients with neuropathic pain vs. healthy volunteers. Data are given as means ± SD; *p < 0.05, **p < 0.01

First, to confirm our previous findings, we determined the Foxp3/CD4^+^ cell ratio in neuropathic pain patients as compared to healthy volunteers. As shown in Fig. 1a, neuropathic pain patients exhibited significantly elevated Foxp3/CD4+ ratios (1.6 ± 0.9 in neuropathic pain vs. 0.8 ± 0.5 in healthy controls; *p* < 0.05). Noteworthy, number of Tregs was not correlated with age (Additional file 1: Figure S1A). Next, we investigated the mRNA expression of SIRT1 in CD4^+^ T cells obtained from patients suffering from neuropathic pain as compared to healthy volunteers. As shown in Fig. 1b, neuropathic pain patients exhibited a markedly reduced SIRT1 mRNA expression (0.5 ± 0.2 in neuropathic pain vs. 1.0 ± 0.4 in healthy controls; *p* < 0.01, Fig. 1b).

### Knockdown of SIRT1 in human CD4^+^ T cells induces Treg differentiation in vitro

**Fig. 2:**
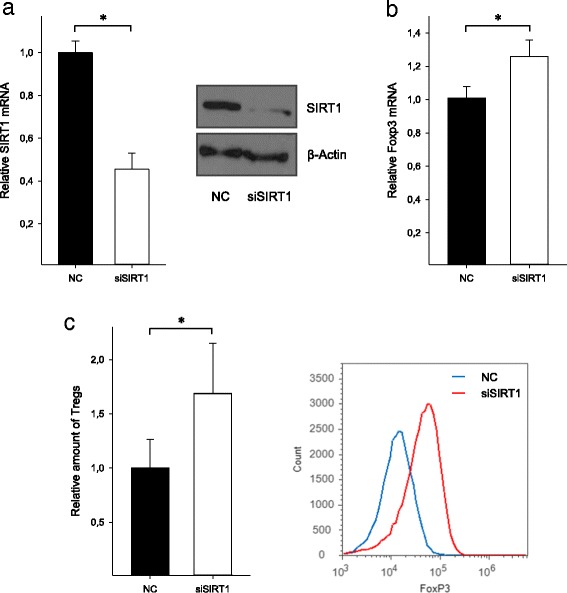
SIRT1 is a negative regulator of Foxp3 expression in human T cells. SIRT1 knockdown in primary human CD4^+^ T cells of healthy volunteers was performed by specific siRNA, and Foxp3 mRNA expression and Treg differentiation was analyzed after incubation under Treg skewing conditions for 4 days. **a** Successful transfection of siRNA was confirmed by qPCR (*left panel*) and Western Blot analysis (*right panel*). One blot is representative of *n* = 3. **b** Relative Foxp3 mRNA expression after transfection and Treg differentiation as measured by qPCR. **c** Treg subpopulation as determined by FACS analysis (*left panel*), a histogram representative of *n* = 6 individual experiments performed in duplicates is shown in the right panel. Data are given as means ± SD; *p < 0.01, *n* = 6

SIRT1 is known as an important negative regulator of Foxp3 expression in murine T cells. To gain insight into its functions in human T lymphocytes, we next analyzed the impact of SIRT1 knockdown in primary human CD4^+^ T cells on Foxp3 expression and Treg differentiation. Transfection of human CD4^+^ T cells with SIRT1 siRNA significantly reduced both SIRT1 mRNA and protein expression (mRNA 0.5 ± 0.1, *n* = 6; *p* < 0.01, Fig. 2a) as compared to normal control (NC). After incubation of transfected and stimulated cells under Treg skewing conditions for 4 days, an increase of Foxp3 mRNA expression by approximately 30 % was found (1.3 ± 0.1, *n* = 3; *p* < 0.01, Fig. 2b). Accordingly, Treg differentiation was clearly enhanced (1.7 ± 0.4, *n* = 3; *p* < 0.01, Fig. 2c). These findings imply that a decreased SIRT1 expression may significantly contribute to the increase of Treg cells in neuropathic pain.

### miR-124a and miR-155 are potential candidates of SIRT1 regulation in neuropathic pain

**Fig. 3:**
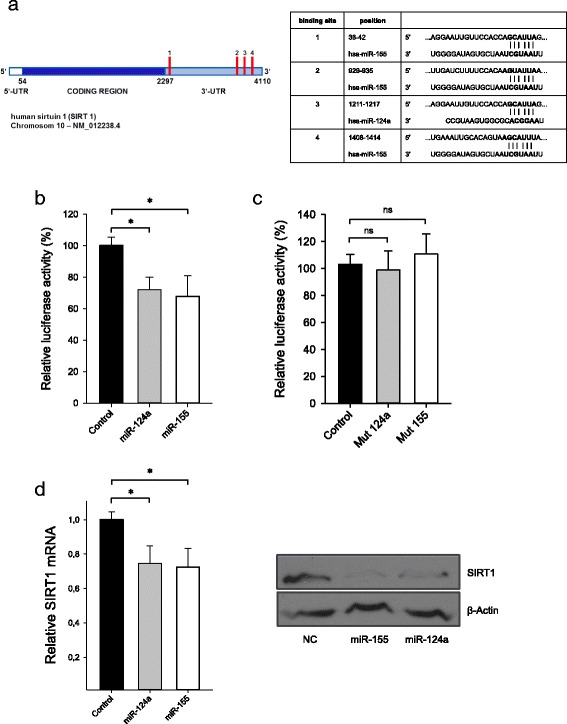
SIRT1 is a novel miR-124a and miR-155 target. Depiction of the genomic structure of the human SIRT1 gene on chromosome 10 and location of the putative miR-124a and miR-155 binding sites within its 3′-UTR (**a**). Target prediction algorithm identified a putative miR-124a binding site and three putative miR-155 binding sites, indicated by the *red bars*. Positions and seed sequences of the putative binding sites are listed in the adjacent table (**a**). A reporter vector containing the SIRT1 3′-UTR was co-transfected with pre-miR-124a or pre-miR-155 into HEK-293 cells, and hRLuc reporter activity was determined relative to a vector construct containing the SIRT1 3′-UTR co-transfected with pre-miR-scrambled control (**b**). Control constructs lacking either the miR-124a (Mut 124a) or the miR-155 binding sites (Mut 155) were generated by site-directed mutagenesis. Both mutant vectors were co-transfected with the respective miRNA or with scrambled control into HEK-293 cells, and hRLuc reporter activity was determined; luciferase activity relative to scrambled control is given. Data are means ± SD; *ns* not significant, *p < 0.01, *n* = 8. **c** CD4^+^ T cells of healthy donors were transiently transfected with miR-124a, miR-155, or scrambled control, respectively, and stimulated with anti-CD3/CD28 Dynabeads for 36 h. Relative SIRT1 mRNA was detected by qPCR, *n* = 6, *p < 0,01 (**d**, *left panel*), and SIRT1 protein expression was determined by Western Blot analysis (**d**, *right panel*). One blot is representative of *n* = 3

We next hypothesized that regulation by specific miRNAs may influence SIRT1 expression and may thus account for the observed alterations of SIRT1 mRNA expression in neuropathic pain. To this end, we combined results of published microarray data in patients with chronic pain syndromes with target prediction in silico. These analyses revealed miR-124a and miR-155 as potential candidates involved in the regulation of SIRT1 in neuropathic pain. Target prediction tools suggested three specific binding sites for miR-155 and a single specific binding side for miR-124a with high probability within the 3′-UTR of the SIRT1 transcript (Fig. 3a).

### SIRT1 expression is directly regulated by miR-124a and miR-155

To provide an experimental proof of a direct interaction between miR-124a and/or miR-155 with the SIRT1 3′-UTR, we performed luciferase reporter assay on a psiCheck-2 plasmid containing a Renilla luciferase gene upstream of the SIRT1 3′-UTR. HEK293 cells were transiently co-transfected with the reporter vector construct and either pre-miR-124a or pre-miR-155 or NC, and luciferase activity was measured. As shown in Fig. 3b, reporter activity was significantly reduced by both miRNAs (miR-124a 72 ± 7 %, miR-155 68 ± 13 %, *n* = 8; *p* < 0.01), as compared to NC.

Site-directed mutagenesis of either the miR-124a or the three miR-155 binding sites within the 3′-UTR of SIRT1 strongly diminished the inhibitory effect of the respective miRNA (Fig. 3c). These data demonstrate that both miRNAs regulate SIRT1 expression by direct targeting of specific binding sites within the 3′-UTR of SIRT1.

Next, we validated the impact of miR-124a and miR-155 on the expression of SIRT1. We assessed SIRT1 mRNA levels after transfection of human CD4^+^ T cells with either miR-124a or miR-155 mimics or with negative control. As depicted in Fig. 3d, SIRT1 mRNA (miR-124a 0.75 ± 0.1, miR-155 0.72 ± 0.1; *n* = 6; *p* < 0.01) and protein expression significantly decreased after transient transfection of both miRNAs as compared to control.

Taken together, we provide evidence that SIRT1 mRNA expression in primary human T cells is directly regulated by miR-124a and miR-155.

### miR-124a and miR-155 control SIRT expression in neuropathic pain

**Fig. 4:**
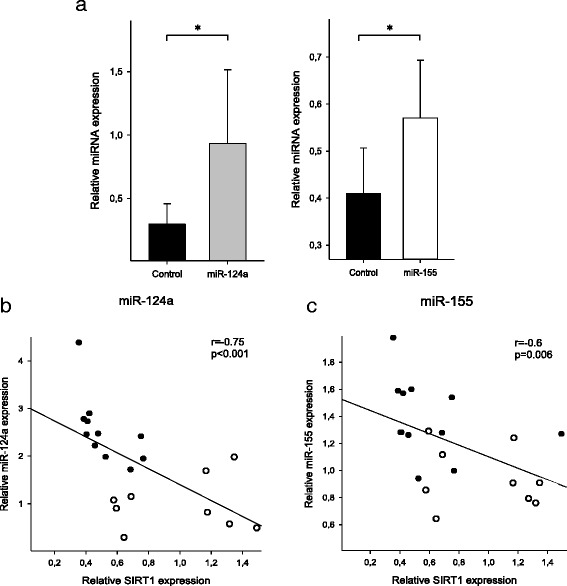
miR-124a and miR-155 are upregulated in CD4^+^ T cells of patients with neuropathic pain. **a** Total RNA was extracted from purified CD4^+^ T cells of patients suffering from neuropathic pain (*n* = 11) as well as from those of healthy volunteers (*n* = 9, control). Relative expression of endogenous miR-124a and miR-155 was quantified by qPCR using U47 RNA as normalizing control. Expression of miR-124a (**a**﻿, left panel) and miR-155 (**a**﻿, right panel) in patients with neuropathic pain as compared to healthy volunteers is shown. Data are given as means ± SD; *p < 0.05. Correlation of miR-124a expression (**b**) and miR-155 expression (**c**) and SIRT1 transcript levels in human CD4^+^ T cells

We next determined the expression of miR-124a and miR-155 in CD4^+^ T cells obtained from patients with neuropathic pain and from healthy volunteers. Expression of both miRNAs was significantly higher in patients with neuropathic pain as compared to healthy volunteers (miR-124 2.5 ± 0.7, *p* < 0.05, Fig. 2a, miR-155 1.3 ± 0.3; *p* < 0.05, Fig. 4a). Correlation analyses in human CD4^+^ T cells revealed for both miRNAs a significant inverse correlation with SIRT1 transcript levels (miR-124a: *r* = −0.75, *p* < 0.001, *n* = 20, miR-155: *r* = −0.6, *p* = 0.006, *n* = 20, Fig. 4b, c), which strongly points to an important role of both miRNAs as regulators of SIRT1 in vivo. There was no significant correlation between age and either miR-124a, miR-155, or SIRT1 mRNA expression (Additional file 1: Figure S1B-D).

### miR-124a and miR-155 increase Treg differentiation

**Fig. 5:**
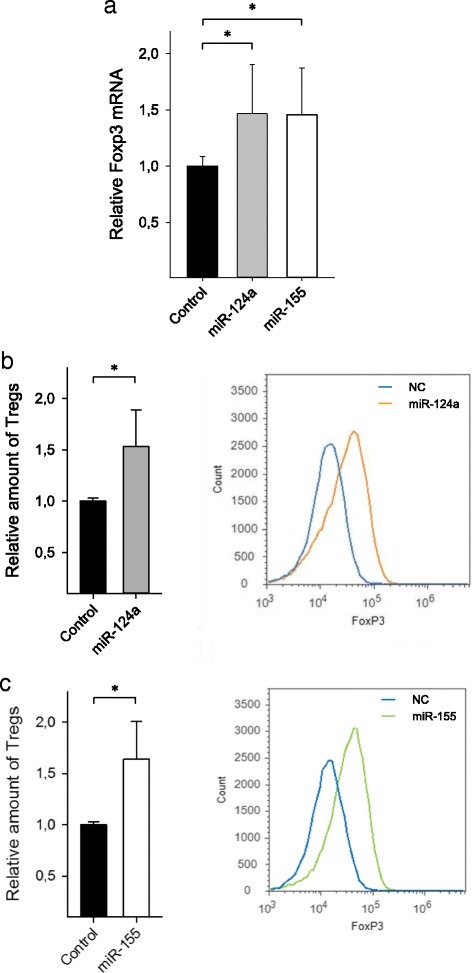
miR-124a and miR-155 enhance Treg differentiation. Human CD4^+^ T cells were transfected with either pre-miR-124a, pre-miR-155, or scrambled control, followed by culturing under Treg skewing conditions for 4 days. Relative Foxp3 mRNA expression was detected by qPCR (**a**). The number of Tregs was determined by FACS analysis (**b**, **c**). Additionally, representative histograms of the FACS analysis are shown in **b** and **c** (*right panels*). Data are given as means ± SD; *p < 0.01, *n* = 6

To investigate the impact of both miRNAs on Treg differentiation, we transfected human CD4^+^ T cells with either pre-miR-124a or pre-miR-155 followed by culturing under Treg skewing conditions for 4 days. As shown in Fig. 5a, Foxp3 mRNA expression was significantly increased in miRNA-transfected cells as compared to controls (miR-124a 1.5 ± 0.4; *n* = 6; *p* < 0.01; miR-155 1.5 ± 0.4; *n* = 6; *p* < 0.01; Fig. 5a). Additionally, expression of the Treg signature molecules EOS, CTLA4, and IL2RA [21] was also elevated (Additional file 1: Figure S2). Accordingly, an enhancement of Treg differentiation was found (miR-124a 1.5 ± 0.4; *n* = 6; *p* < 0.01; miR-155 1.6 ± 0.4; *n* = 6; *p* < 0.01; Fig. 5b, c). In accordance with these in vitro findings, we found a significant correlation between both miR-124a and miR-155 expression and Foxp3 mRNA expression in human CD4^+^ T cells (Additional file 1: Figure S3). These findings demonstrate an impact of miR-124a and miR-155 on Treg differentiation via targeting of SIRT1.

## Discussion

The pathophysiology of neuropathic pain is not fully understood. Recent studies have established proof that aberrant responses of the adaptive immune system substantially contribute to the development of this clinical disorder. Underlying mechanisms, however, are largely unknown. In this study, we show an involvement of miRNAs in the regulation of inflammatory processes in neuropathic pain. We identify miR-124a and miR-155 as direct repressors of the deacetylase SIRT1. Targeting of SIRT1 by these miRNAs results in an increase of Foxp3 expression and, consecutively, of anti-inflammatory Tregs. We here show that in patients suffering from neuropathic pain as compared to healthy volunteers, an increased expression of miR-124a and miR-155 inhibits SIRT1 expression, which enhances CD4^+^ T cell differentiation towards Tregs.

Peripheral nerve injury leads to the release of factors that recruit and activate immune cells from the circulation. These cells secrete pro-inflammatory mediators that contribute to the development of pain symptoms. In particular, the T cell response is considered an important contributor to the development of neuropathic pain. In animal models of peripheral nerve injury, pain sensitivity of T cell deficient animals was significantly attenuated, which could be restored by adoptive transfer of pro-inflammatory cytokine producing Th1 cells [8]. On the other hand, expansion of Tregs, which limit immune responses of pro-inflammatory T cells, led to a significant reduction of pain hypersensitivity [22] while depletion of Tregs promoted pain hypersensitivity by inducing altered systemic concentrations of cytokines in mice [6]. The latter findings point towards a possible role of Tregs in the limitation of pain promoting inflammatory responses. In a very recently published study, we reported an increase of the Treg subpopulation in the peripheral blood of patients suffering from neuropathic pain, which also points into that direction. The pathways leading to the observed Treg induction, however, have not been addressed yet.

We here suggest a decreased expression of the histone-deacetylase SIRT1 as a possible underlying mechanism. SIRT1 is known to control Treg differentiation and function (i) by promoting Foxp3 gene expression and (ii) by Foxp3 lysine ε-aminodeacetylation leading to ubiquitination and proteasomal degradation. Here, we show that targeting of SIRT1 by specific siRNA promotes Treg differentiation of human CD4^+^ T cells in vitro. Similar results have been found in a recently published study, which reported an increased differentiation of naive T cells to Tregs after treatment with SIRT1 inhibitors in mice. Our findings strongly suggest that a decrease of SIRT1 expression contributes to the observed increase of Treg cells in neuropathic pain patients.

SIRT1 is subject to regulation on a transcriptional and posttranscriptional level [23, 24]. Particularly in tumors and endothelial cells, miRNAs have been shown to influence SIRT1 expression (e.g., miR-29c [23], miR-141 [23], miR-200 [24], miR-204 [25]). Based on the assumption that alterations of miRNA profiles might also be involved in the regulation of SIRT1 in neuropathic pain, we focused on miRNAs as potential suppressors of SIRT1 in this context. We identified miRNA-124a and miRNA-155 as potential candidates binding to the SIRT1-3′-UTR with high probability in silico. miR-155 is expressed in multiple types of immune cells and has been proposed to affect a wide range of immunological processes under physiologic conditions as well as in the course of immune responses [26–31]. In mice studies, it has been shown that the expression of miR-155 in Treg cells is required to maintain normal Treg numbers and function, which was in part attributed to miR-155-mediated SOCS1 repression [32, 33]. In a rat model of neuropathic pain, inhibition of miR-155 was shown to reduce cytokine production of microglial cells via SOCS1 repression, thereby attenuating pain symptoms [34].

miR-124a is predominantly expressed in the central nervous system (CNS). There, it displays specific temporal and spatial expression profiles in various cell types and affects a variety of biological functions. Dysregulation of miR-124 has been linked to several pathologic conditions of the CNS, such as brain tumors, neurodegeneration, epilepsy, and neuroimmune disorders. Furthermore, miR-124a is involved in macrophage polarization, which impacts a variety of diseases. For example, in animal models of pain, intrathecal application of miR-124a resulted in a decrease of pro-inflammatory cytokines secreted by microglia/macrophages, which led to a reduction of persistent hyperalgesia [35, 36].

Roads of miRNA regulation are redundant and highly dependent on the cellular and physiological context. Here, we reveal a new function of miR-124a and miR-155 in T cells in neuropathic pain: Our experiments show that both miRNAs suppress SIRT1 mRNA expression by direct targeting of specific binding sites. Accordingly, overexpression of miR-124a and miR-155 in human CD4^+^ T cells in vitro suppressed SIRT1 and, in accordance with our in vitro results obtained by transfection of SIRT1 siRNA, induced a bias towards Treg differentiation.

Clinical data also support this hypothesis: In T cells of neuropathic pain patients, we detected an increased expression of miR-124a and miR-155. Moreover, the expression of these two miRNAs was inversely correlated with SIRT1 transcript levels, which strongly supports the hypothesis that the Treg shift observed in neuropathic pain, indeed, is at least partially driven by a miRNA-mediated mechanism.

## Conclusions

Increasing peripheral Treg numbers may be an endogenous attempt to limit inflammation, thus reducing pain levels in neuropathic pain. We here demonstrate that lymphocytic miRNAs significantly contribute to these adaptive processes. Deciphering miRNA-target interactions that influence inflammatory pathways in neuropathic pain may thus help to develop new approaches of pain amelioration.

## Additional file

Additional file 1: Figure S1. Correlation analysis of age and (A) Treg numbers, (B) SIRT1 mRNA, (C) miRNA-124 expression, and (D) miR-155 expression. Analyses revealed no significant correlations. Black dots: Neuropathic pain patients, white dots: Healthy volunteers. Figure S2 Human CD4 + T cells were transfected with either pre-miR-124a, pre-miR-155, or scrambled control, followed by culturing under Treg skewing conditions for 4 days. Relative mRNA expression the Treg signature molecules EOS, CTLA4, and IL2RA was detected by qPCR; **p* < 0.05, ***p* < 0.01, *n* = 5. Figure S3. Correlation analysis of either miR-124a (A) or miR-155 (B) and Foxp3 mRNA expression. Black dots: Neuropathic pain patients, white dots: Healthy volunteers. (PPTX 227 kb)

## Abbreviations

Tregs: Regulatory T cells
SIRT1: Histone deacetylase sirtuin1
Foxp3: Transcription factor forkheadbox-p3
NeP: Neuropathic pain
CRP: C-reactive protein
NRS: Numerical rating scale
PBMCs: Peripheral blood mononuclear cells
qPCR: Quantitative real-time PCR (RT-PCR)
FACS: Fluorescent-activated cell sorting
KAB: Kurzfragebogen zur aktuellen Beanspruchung
SDHA: Succinate dehydrogenase complex subunit A
TBP: TATA box binding protein
SD: Standard deviation
CNS: Central nervous system
BMI: Body mass index
