# Supplementary material for: miR-124a and miR-155 enhance differentiation of regulatory T cells in patients with neuropathic pain
Source: J Neuroinflammation. 2016 Sep 20;13:248. doi: 10.1186/s12974-016-0712-6 (PMC5029065; doi:10.1186/s12974-016-0712-6)
Supplement: Additional file 1: Figure S1. — Correlation analysis of age and (A) Treg numbers, (B) SIRT1 mRNA, (C) miRNA-124 expression, and (D) miR-155 expression. Analyses revealed no significant correlations. Black dots: Neuropathic pain patients, white dots: Healthy volunteers. Figure S2 Human CD4 + T cells were transfected with either pre-miR-124a, pre-miR-155, or scrambled control, followed by culturing under Treg skewing conditions for 4 days. Relative mRNA expression the Treg signature molecules EOS, CTLA4, and IL2RA was detected by qPCR; *p < 0.05, **p < 0.01, n = 5. Figure S3. Correlation analysis of either miR-124a (A) or miR-155 (B) and Foxp3 mRNA expression. Black dots: Neuropathic pain patients, white dots: Healthy volunteers. (PPTX 227 kb) [file 12974_2016_712_MOESM1_ESM.pptx]

## Slide 1
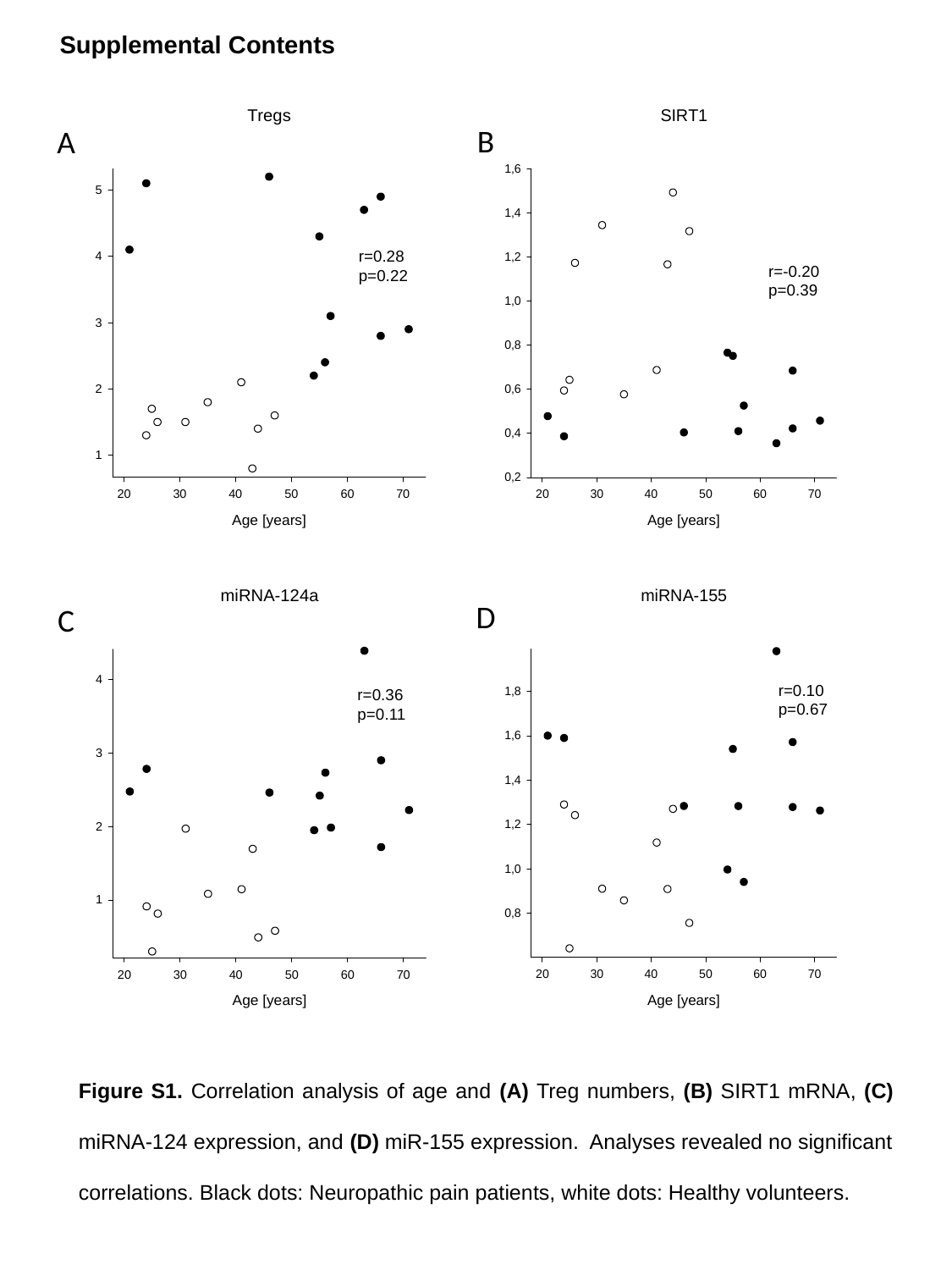

Supplemental Contents
r=0.28
p=0.22
r=-0.20
p=0.39
B
A
r=0.10 p=0.67
r=0.36
p=0.11
D
C
Figure S1. Correlation analysis of age and (A) Treg numbers, (B) SIRT1 mRNA, (C) miRNA-124 expression, and (D) miR-155 expression. Analyses revealed no significant correlations. Black dots: Neuropathic pain patients, white dots: Healthy volunteers.

## Slide 2
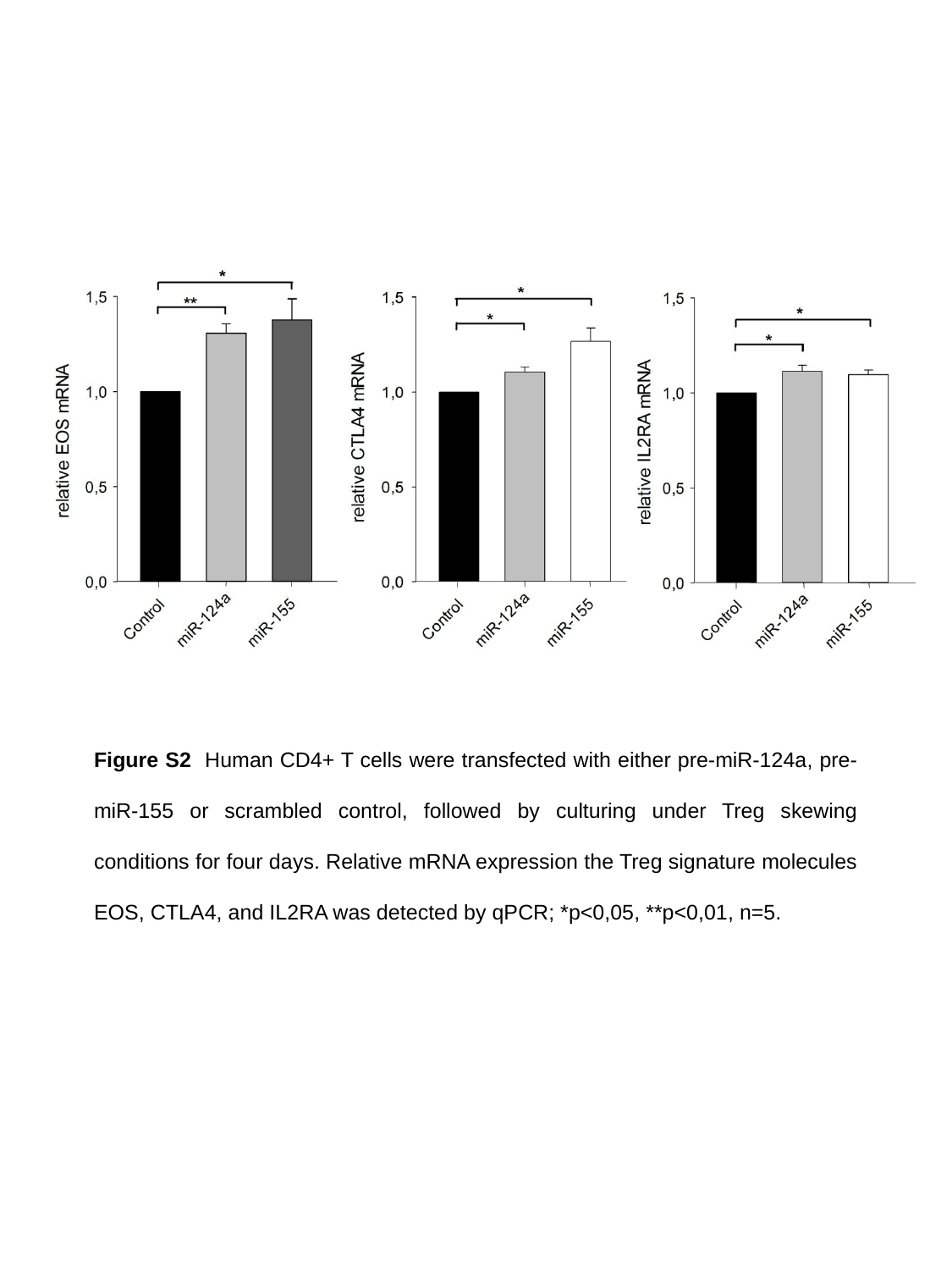

Figure S2 Human CD4+ T cells were transfected with either pre-miR-124a, pre-miR-155 or scrambled control, followed by culturing under Treg skewing conditions for four days. Relative mRNA expression the Treg signature molecules EOS, CTLA4, and IL2RA was detected by qPCR; *p<0,05, **p<0,01, n=5.

## Slide 3
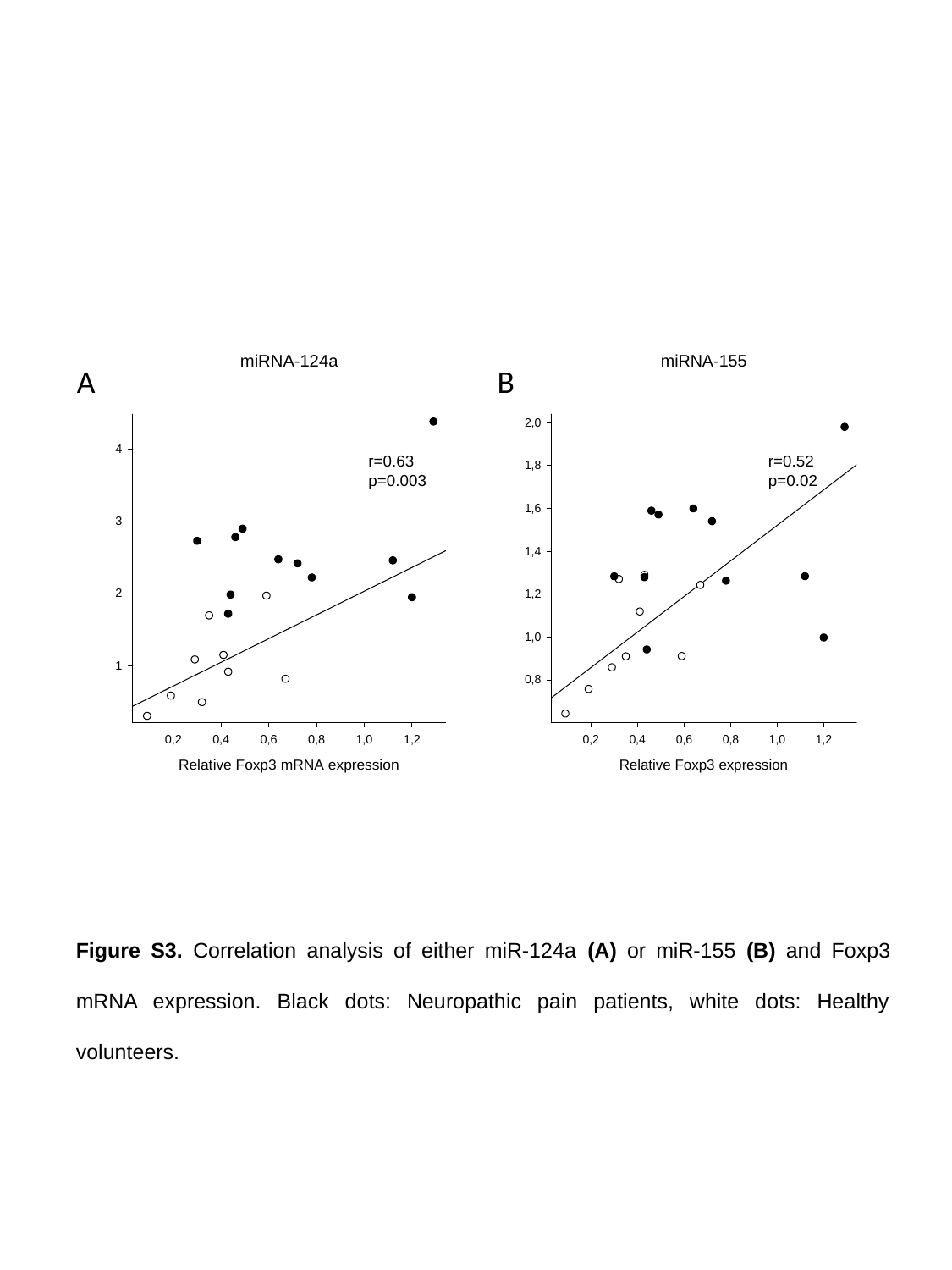

B
A
r=0.63
p=0.003
r=0.52
p=0.02
Figure S3. Correlation analysis of either miR-124a (A) or miR-155 (B) and Foxp3 mRNA expression. Black dots: Neuropathic pain patients, white dots: Healthy volunteers.
